# Dangerous Byproducts of Alcohol Breakdown— Focus on Adducts

**Published:** 2003

**Authors:** Dean J. Tuma, Carol A. Casey

**Affiliations:** Dean J. Tuma, Ph.D., is a senior research career scientist and director of the Omaha VA Alcohol Research Center, Department of Veterans Affairs. He also is a professor in the Department of Internal Medicine and the Department of Biochemistry and Molecular Biology at the University of Nebraska Medical Center, Omaha. Carol A. Casey, Ph.D., is a research career scientist in the Department of Veterans Affairs and an associate professor in the Department of Internal Medicine and the Department of Biochemistry and Molecular Biology at the University of Nebraska Medical Center, Omaha

**Keywords:** ethanol metabolism, adduct, aldehydes, acetaldehyde, oxygen radicals, alcoholic liver disorder, proteins, immune system, toxic drug effect, lipids, peroxidation, biochemical mechanism

## Abstract

Alcohol breakdown in the liver results in the generation of the reactive molecule acetaldehyde and, as a byproduct, highly reactive oxygen-containing molecules known as oxygen radicals. Both acetaldehyde and oxygen radicals can interact with proteins and other complex molecules in the cell, forming hybrid compounds called adducts. Other adducts are formed with aldehyde molecules, which are produced through the interaction of oxygen radicals with lipids in the cells. Adduct formation impedes the function of the original proteins participating in the reaction. Moreover, the adducts may induce harmful immune responses. Both of these effects may account for some of the damage observed in alcoholic liver disease. Adduct formation has been shown to occur in the livers of humans and animals consuming alcohol and to start and predominate in those liver regions that show the first signs of liver damage.

Most of the alcohol a person ingests is eliminated from the body via a series of chemical reactions in the liver that are collectively referred to as oxidative metabolism. Because the liver is one of the organs that most commonly exhibits alcohol-induced damage, researchers have attributed many of the disturbances in liver structure and function frequently seen in alcoholics to the products of alcohol metabolism (Lieber 1988; Tuma and Sorrell 1995). The most important enzyme involved in the breakdown of alcohol is called alcohol dehydrogenase, which converts alcohol into acetaldehyde, a highly reactive and toxic molecule that may play a crucial role in alcohol-related liver damage.

Another enzyme that can mediate the initial step of alcohol metabolism is cytochrome P450 2E1. The chemical reaction promoted by this enzyme also results in the formation of acetaldehyde, as well as in the production of highly reactive oxygen-containing molecules called oxygen radicals, including the hydroxyethyl radical (HER). Excessive production of oxygen radicals and/or a concurrent deficiency of molecules that can eliminate these radicals (i.e., antioxidants) creates a condition in the cell known as oxidative stress, which can lead to cell death. (For more information on the role of oxygen radicals and oxidative stress in liver disease, see the article in this issue by Wu and Cederbaum.) Furthermore, oxygen radicals can interact with fat (i.e., lipid) molecules in the cell membranes in a process called lipid peroxidation, which in turn results in the generation of additional reactive molecules similar to acetaldehyde, especially malondialdehyde (MDA) and 4-hydroxy-2-nonenal (HNE) (Cederbaum 2001; Niemela 1999).

Because acetaldehyde and the lipid peroxide–derived aldehydes (i.e., MDA and HNE) are chemically reactive, they can interact with proteins and other complex molecules to form modified molecules known as adducts. The formation of these aldehyde–protein adducts is believed to be a key event in the development of alcohol-induced liver injury (Niemela 2001; Tuma and Sorrell 1995; Tuma 2002).

This article describes the types of adducts formed in the liver, reviews evidence that such adducts are generated during alcohol consumption, and discusses the possible role that adducts may play in liver injury.

## Types of Adducts Formed in the Liver During Alcohol Consumption

As mentioned above, alcohol degradation using both alcohol dehydrogenase and cytochrome P450 2E1 generates reactive compounds that can bind to proteins and form adducts (see the accompanying figure). Numerous studies have reported that a variety of protein adducts are formed in the liver as a result of alcohol consumption and degradation. The major reactive molecules participating in adduct formation appear to be those that are chemically known as aldehydes, specifically acetaldehyde, MDA, and HNE (Niemela 2001; Tuma and Sorrell 1995).

**Figure:**
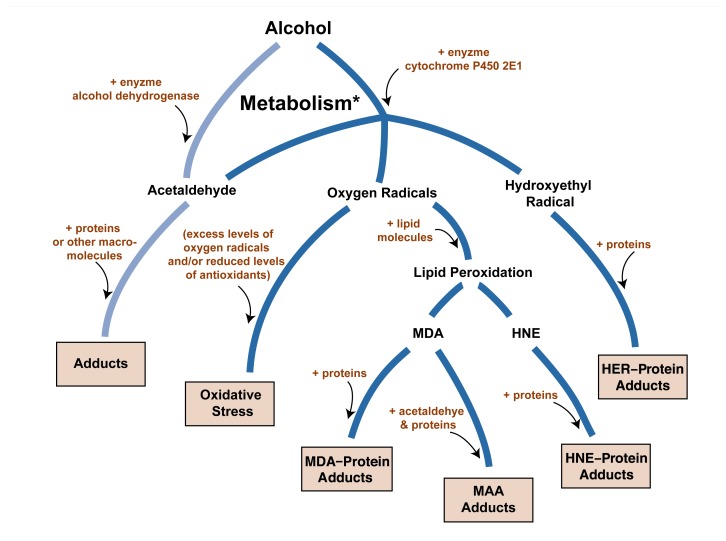
Potentially toxic products resulting from the breakdown, or metabolism, of alcohol (chemically known as ethanol). The major alcohol-metabolizing enzymes are alcohol dehydrogenase and cytochrome P450 2E1 (CYP2E1). Alcohol dehydrogenase converts alcohol to acetaldehyde, which can react with other proteins in the cell to generate hybrid molecules known as adducts. CYP2E1 also generates acetaldehyde, as well as highly reactive oxygen-containing molecules called oxygen radicals, including the hydroxyethyl radical (HER) molecule. Elevated levels of oxygen radicals can generate a state of oxidative stress, which through various mechanisms leads to cell damage. Oxygen radicals also can interact with fat molecules (lipids) in the cell in a process known as lipid peroxidation, resulting in reactive molecules such as malondialdehyde (MDA) and 4-hydroxy-2-nonenal (HNE). Both of these can react with proteins to form MDA–protein and HNE–protein adducts. MDA also can combine with acetaldehyde and protein to form mixed MDA–acetaldehyde–protein adducts (MAA). HER also interacts with protein to form HER–protein adducts. * Also referred to as “breakdown,” “oxidation,” and “degradation.”

The formation of acetaldehyde–protein adducts has received the most attention from researchers to date (Tuma and Sorrell 1995; Worrall and Thiele 2001). Acetaldehyde can react with proteins in the body to form both unstable adducts, which are immediately converted into other compounds, and stable adducts, which remain in the cell for a certain length of time. Proteins are made up of approximately 20 different building blocks (i.e., amino acids). Researchers have found that acetaldehyde interacts with specific amino acids, particularly lysine, during adduct formation. Although investigators also have identified the chemical group—a so-called amino group^1^—with which acetaldehyde interacts, the exact chemical structures of the stable adducts have not yet been resolved.

**Glossary of Abbreviations**

HER: Hydroxyethyl radical, a highly reactive oxygen radical that participates in adduct formation
HNE: 4-hydroxy-2-nonenal, a reactive molecule that is generated during lipid peroxidation and participates in adduct formation
MAA: Mixed MDA–acetaldehyde–protein adducts; compounds that contain MDA plus acetaldehyde plus protein components
MDA: Malondialdehyde, a reactive molecule that is generated during lipid peroxidation and participates in adduct formation.

The lipid peroxide–derived aldehydes, MDA and HNE, also can react with proteins, generating a variety of adducts (Esterbauer et al. 1991; Worrall and Thiele 2001). MDA, like acetaldehyde, reacts mainly with amino groups found in proteins and forms a variety of diverse adducts, complicating the analysis of the exact processes occurring during MDA–protein adduct formation. HNE can react with various amino acids in proteins but appears to interact primarily with the amino acids lysine, cysteine, and histidine to form relatively stable HNE–protein adducts.

### Formation of Mixed (Hybrid) Adducts

Each of the aldehydes discussed here can form adducts with proteins on its own. During alcohol metabolism in the liver, however, the aldehydes coexist in the cells and may influence each other’s reactivity with proteins. For example, researchers have found that MDA and acetaldehyde react with proteins in a synergistic manner (Tuma et al. 1996; Tuma 2002). This means that the presence of both aldehydes strikingly increases each aldehyde’s binding to proteins, generating mixed (i.e., hybrid) adducts that clearly differ from the adducts formed by either aldehyde alone. These composite MDA–acetaldehyde–protein adducts, called MAA adducts, consist of two major components whose structures have been determined and that contain the amino acid lysine: (1) a relatively stable compound containing two molecules of MDA and one molecule of acetaldehyde; and (2) a compound containing one molecule of MDA and one molecule of acetaldehyde, which appears to serve as a precursor for the more stable adduct.

### Proteins That Are Targets of Aldehyde Adduct Formation

Although many proteins contain the amino acids—primarily lysine—that interact with the aldehydes, not all of those proteins are equally likely to form adducts with aldehydes. Proteins that appear to be preferentially modified by aldehydes include, among others, the following (Niemela 1999):

- Hemoglobin, which is crucial for the transport of oxygen in the red blood cells.
- Albumin, a protein found in the blood.
- Tubulin, a component of cell structures called microtubules that are essential for cell division as well as for the secretion and transport of proteins within the cells.
- Lipoproteins, which consist of proteins and fat molecules and influence the risk of heart disease.
- Collagen, the major protein in the connective tissues, which is involved in scar tissue formation.
- Cytochrome P450 2E1, which plays a role in the breakdown of alcohol and other toxic substances.
- Ketosteroid reductase, a key enzyme involved in proper digestion (i.e., it influences the production of bile acid biosynthesis).

Researchers have not yet determined the basis for this preferential binding of aldehydes to certain proteins. It seems, however, that some lysine residues in proteins are particularly reactive with aldehydes and preferentially form adducts because of their local environments (i.e., the other amino acids surrounding them within the protein) (Tuma and Sorrell 1995).

### Other Types of Adducts

Aldehyde–protein adducts are not the only class of adducts formed during alcohol metabolism; another class of adducts involves oxygen radicals generated during alcohol breakdown. For example, cytochrome P450 2E1–mediated oxidation of alcohol can result not only in the formation of acetaldehyde but also in the formation of the HER oxygen radical that can readily bind to proteins and form HER–protein adducts. Because it is highly reactive, HER is likely to react with a variety of different sites on proteins (Moncada and Israel 2001; Worrall and Thiele 2001), generating a range of adducts.

Adducts created with proteins are the most widely studied and probably the most important adducts resulting from alcohol metabolism. Nevertheless, the aldehydes and oxygen radicals generated during alcohol metabolism also can form adducts with other complex molecules, such as DNA or lipids. Further study of the roles and consequences of these adducts is needed.

## In Vivo Formation of Adducts During Alcohol Consumption

The fact that it is possible to create adducts through chemical reactions in test tubes (i.e., in vitro) does not prove that adduct formation actually occurs in the liver (i.e., in vivo) and contributes to the development of alcoholic liver disease (ALD). Therefore, it is important to verify that adducts are generated in the liver during alcohol consumption. Numerous studies over the years have indicated that chronic alcohol consumption in both animals and humans results in the formation of various protein adducts in the liver; the findings of these studies are summarized in this section.

The first evidence indicating that adducts are generated in vivo as a result of alcohol intake came from studies demonstrating that after proteins have been modified by reactive molecules such as aldehydes, the adducts that are produced can elicit an immune response (Niemela 2001). This means that the body recognizes these adducts as something “foreign” (i.e., not normally belonging to the body) and in response generates antibodies that recognize and bind to these foreign molecules. The interaction between a foreign molecule and the antibodies produced by the body marks the foreign molecule for destruction by other immune cells. In most cases, however, the antibodies recognize the modified protein adducts but not the original proteins; therefore, the original proteins can remain in the body and exert their influence without being destroyed by the immune system.

Researchers subsequently have detected antibodies against acetaldehyde, MDA, HNE, MAA, and HER adducts in the blood of animals and humans who chronically consumed alcohol, implying that adducts are formed in the body and then stimulate the production of antibodies (Clot et al. 1997; Niemela 2001; Tuma 2002; Worrall and Thiele 2001). These findings provide indirect evidence that alcohol metabolism in vivo does indeed result in the formation of aldehyde– protein and HER–protein adducts.

Direct evidence that adducts form in the livers of animals and humans consuming alcohol was derived from studies using antibodies against specific adducts. For example, several studies (Niemela 2001; Worrall and Thiele 2001) have reported the presence of acetaldehyde–protein adducts in the livers of alcohol-fed animals and humans. These adducts were detected mainly in the principal liver cells (i.e., the hepatocytes), although they may occur in other types of liver cells as well. Various other types of adducts also have been observed, sometimes at specific locations in the liver or liver cells, as follows:

- Most of the proteins that are modified by acetaldehyde appear to be located in the fluid filling the cells (i.e., the cytosol); however, adducts also form with proteins embedded in the membrane surrounding the cells and in other membrane-enclosed cell structures. Acetaldehyde adducts are found predominantly in a certain region of the liver lobules^2^ (i.e., the perivenous region, which is located around the small vein through which the blood exits the lobule).
- MDA and HNE adducts have been observed in the livers of alcohol-consuming animals; like acetaldehyde adducts, MDA adducts are found at sites in the liver where tissue damage can be detected.
- MAA adducts are generated in vivo and appear to involve primarily proteins in the cytosol of liver cells (Tuma et al. 1996; Tuma 2002).
- HER adducts have been detected in the cell membrane and in certain membrane-enclosed cell components (i.e., microsomes) of the hepatocytes of alcohol-fed animals (Clot et al. 1997; Niemela 2001).

## Adduct Formation and Its Role in Liver Injury

Although the formation of adducts in the liver during alcohol consumption has been well established, more information is needed concerning the effects of these adducts on liver cell function and the role they play in liver injury. Recent research in this area has provided some interesting and exciting information on the link between adduct formation and liver dysfunction and injury. These findings may represent a first step in the development of therapeutic interventions that can interfere with adduct formation and its consequences and thereby help reduce the risk of ALD.

One line of evidence concerns the locations of adduct formation and alcohol-related tissue damage in the liver. As mentioned in the previous section, acetaldehyde adducts form primarily in the perivenous region of the liver, which is also the region where alcoholic liver injury starts and predominates, thus supporting the hypothesis that acetaldehyde adducts may contribute to alcoholic liver disease. Moreover, acetaldehyde adducts are evident in the early phase of ALD, and in advanced liver disease they are found in the same areas that show evidence of inflammation and scar tissue formation (i.e., fibrosis). MDA adducts are found at the same sites as (i.e., colocalize with) acetaldehyde adducts in areas where changes in tissue structure (i.e., histological changes) occur in alcoholic liver disease (Niemela 1999, 2001). Additional studies using a specially bred type of small pig (i.e., micropigs) as a model system for the development of alcoholic liver disease reported a progressive accumulation of acetaldehyde, MDA, and HNE adducts after prolonged alcohol intake, which coincided with progressive liver injury (Halsted et al. 1993; Niemela et al. 1995). Moreover, acetaldehyde and MDA adducts, which increased after alcohol feeding, colocalized with the sites of collagen deposits, a characteristic step in scar tissue formation that occurs prior to fibrosis. Thus, these findings indicate a link between acetaldehyde and MDA adducts and the subsequent development of fibrosis in the perivenous region.

Researchers have identified several mechanisms through which various adducts could contribute to liver damage. As mentioned earlier, aldehydes interact primarily with the amino acid lysine. Thus aldehydes particularly interfere with the functions of those proteins that carry a lysine residue at a location in the protein that is critical to the protein’s function (Tuma and Sorrell 1995). Examples of such proteins include the lysine-dependent enzymes, the regulatory protein calmodulin, and the cytoskeletal protein tubulin. Acetaldehyde–tubulin adducts appear to be especially important and relevant to alcohol-induced liver injury. Studies have shown that modification of only 5 percent of the individual molecules of a certain type of tubulin (i.e., α-tubulin) by acetaldehyde leads to complete inhibition of tubulin assembly into microtubules (Tuma and Sorrell 1995; Tuma et al. 1991). Impaired microtubule function likely accounts for the observed defects in protein secretion and other protein transport pathways in the liver that result from chronic alcohol consumption (Tuma and Sorrell 1995; Tuma et al. 1991). This altered microtubule function also could lead to a considerable disorganization of the hepatocytes that is characterized by various structural changes and which could progress to more severe liver damage in alcohol abusers. Investigators have described impaired function of numerous proteins other than tubulin by adduct formation; however, the role of these adducts in liver dysfunction and injury remains to be established.

Another important process in the liver that is affected when adduct formation interferes with the functions of proteins is extracellular matrix production. The extracellular matrix is a set of proteins (e.g., collagen, fibronectin, and laminin) that form a kind of scaffolding in which the cells are embedded. Disturbances in extracellular matrix production could lead to the formation of scar tissue in the liver (i.e., hepatic fibrosis)—the abnormal accumulation of extracellular matrix components that is characteristic of stages of alcoholic liver disease prior to cirrhosis. Many studies have shown that acetaldehyde as well as HNE and MDA increase collagen production in various types of liver cells, such as fibroblasts and activated stellate cells^3^ (Niemela 2001; Tuma and Sorrell 1995). The intriguing possibility that adduct-stimulated collagen production may contribute to the overall process of alcoholic hepatic fibrosis has not been completely evaluated.

Another interesting mechanism by which adducts could induce liver injury involves the immune system (Klassen et al. 1995; Niemela 2001; Worrall and Thiele 2001). Numerous studies have shown that aldehyde–protein and HER–protein adducts elicit a distinct immune response, and additional analyses have demonstrated that antibodies against such adducts are present in both humans and animals following chronic alcohol exposure. These findings have led to the hypothesis that adducts formed as a result of alcohol consumption may be recognized by the immune system and may trigger harmful immune responses that could lead to liver damage. This hypothesis is supported by findings that hepatitis can be induced in guinea pigs by feeding alcohol to animals previously immunized with acetaldehyde adducts (Yokoyama et al. 1993). Further studies are necessary to evaluate the exact roles of adducts and the immune system in the development of alcohol-induced liver injury.

### Adverse Effects of MAA Adducts

Recently, researchers also have devoted considerable attention to the role of the two main MAA adducts in liver injury.^4^ Acetaldehyde and MDA can interact with each other and then modify other proteins to form MAA adducts, and conditions in the liver during chronic alcohol metabolism should actually favor the formation of those hybrid MAA adducts over the formation of the individual acetaldehyde and MDA adducts. Therefore, MAA adducts may well represent the most prevalent aldehyde adducts formed in the liver after alcohol consumption.

### Effects Involving the Immune System

MAA adducts elicit a potent immune response that may represent an important event in the development of alcoholic liver injury. Several lines of evidence are consistent with this hypothesis (Thiele et al. 2001; Tuma 2002):

- MAA adducts induce strong antibody responses against certain components of the MAA adducts. Unlike most antibody responses, however, these antibodies sometimes also recognize certain regions of the original proteins, even protein regions that normally identify the protein as belonging to the body and which therefore should not cause an immune response. An immune response to proteins that belong to the body resulting in the destruction of those normal proteins, however, can be highly detrimental to the organism.
- Antibodies against MAA adducts circulating throughout the body have been observed in alcohol-fed animals and in humans with alcoholic liver disease; moreover, the levels of these antibodies correlated with the severity of liver injury.
- Circulating antibodies to “self ” proteins, which probably were generated when these proteins were involved in adduct formation, have been reported in alcohol-fed animals.
- MAA modification of a certain protein found in the liver elicited a specific type of immune response.

Taken together, these findings suggest that the formation of MAA–protein adducts generates harmful compounds that could trigger destructive immune responses targeting liver cells; however, further studies are needed to elucidate this proposed mechanism.

#### Direct Effects

Other investigators have demonstrated that MAA adducts can directly induce processes that promote inflammation and fibrosis in two types of liver cells—the previously mentioned stellate cells and the endothelial cells that line the walls of the small channels that allow blood to flow through the liver (Thiele et al. 2001; Tuma 2002). Exposure of these two cell types to MAA adducts in vitro caused:

- Increased secretion of messenger molecules—cytokines and chemokines—that mediate communication among cells and promote inflammation, including, for example, a cytokine called tumor necrosis factor.
- Increased production of molecules that allow cells to adhere to each other (i.e., cellular adhesion molecules).
- Increased production of a molecule (i.e., fibronectin) that is part of the extracellular matrix and is involved in fibrosis.
- Enhanced secretion of a protein^5^ that leads to the activation of growth factors which promote the formation of tissue fibers (e.g., during scar tissue formation).

All these effects appear to be mediated by MAA adducts that bind to specific molecules (i.e., receptors) on the surface of the liver cells and initiate a cascade of chemical signals within the cells, resulting in altered cellular functions.

To establish the importance of MAA adducts in the development and/or progression of alcoholic liver injury, researchers must conduct similar work in intact organisms (i.e., in vivo) to confirm the results of these in vitro studies indicating that MAA adducts can induce cellular responses that favor inflammation and fibrosis.

## Summary and Perspectives

Excessive alcohol consumption results in the production of several types of protein adducts in the liver. Some plausible and intriguing mechanisms have been proposed to explain the role of adducts in the development of alcoholic liver injury; however, innovative approaches to better identify the mechanisms through which adducts cause liver injury remain challenging goals. The development of new therapeutic interventions for patients with alcoholic liver disease aimed at modifying or preventing adduct formation also poses a challenge for investigators. Both of these endeavors represent fertile areas for future research and should provide valuable information concerning alcohol’s toxic effects on the liver and the treatment of alcoholic liver disease.

## Footnotes

1 An amino group is a structural characteristic of all amino acids.

2 Liver lobules are the functional units of the liver; a human liver consists of approximately 50,000 to 100,000 lobules.

3 Fibroblasts are cells in the connective tissues that can form collagen fibers; stellate cells are fat-storing liver cells whose activation is a crucial step in the development of fibrosis.

4 These compounds are 4-methyl-1,4-dihydropyridine-3,5-dicarbaldehyde (or MDHDC adduct) and 2-formyl-3-(alkylamino) butanal (or FAAB adduct).

5 This protein is urokinase-type plasminogen activator.
